# Micro-Stress Support-Enhanced Two-Plate Shearing Absolute Testing for Φ800 mm Interferometers

**DOI:** 10.3390/s26030858

**Published:** 2026-01-28

**Authors:** Zijia Zhao, Zhiliang Zhao, Yuegang Fu, Jiake Wang, Zhihua Zhang, Yehao Zhao

**Affiliations:** 1 School of Optoelectronic Engineering, Changchun University of Science and Technology, Changchun 130022, China; zijiazhaomk@163.com (Z.Z.); wangjk5013@163.com (J.W.); 2 Chengdu Tyggo Photo-Electricity Co., Ltd., Chengdu 611138, China; zzl_caep@163.com (Z.Z.); zhangzhihua076@163.com (Z.Z.); yehaozhao00@gmail.com (Y.Z.); 3 School of Aeronautics and Astronautics, Xihua University, Chengdu 610039, China

**Keywords:** interferometer, large aperture optical flat, absolute testing

## Abstract

Large-aperture optical elements are increasingly in demand for applications in astronomy, high-power lasers, and aerospace technology, but their manufacturing and testing processes pose significant challenges. In this paper, we propose an ultra-large-aperture digital laser plane interferometric testing technique that combines the two-plate shearing absolute mutual testing method with micro-stress support technology. This method enables high-precision testing of Φ800 mm planar elements and offers advantages such as fast testing speed, high resolution, and precise alignment. Simulation results and comparisons with measurements from a ZYGO interferometer validate the effectiveness of the proposed method. Experimental testing of an Φ800 mm planar element yielded a PV value of 0.0923λ and an RMS value of 0.0114λ at a wavelength of 632.8 nm. The quantitative results are incorporated into the abstract and conclusions, highlighting the method’s minimal error and high accuracy. This technique provides a novel approach for high-precision testing of large-aperture optical elements.

## 1. Introduction

In recent years, with the rapid development of astronomy, high-power lasers, and aerospace technology [1,2], large-aperture optical elements have found widespread applications due to their ability to expand the field of view, improve spatial resolution, and enhance signal collection capabilities [3,4]. This, in turn, has posed higher requirements for their manufacturing processes, where ensuring the uniformity and surface precision of optical elements has become a pressing issue [5]. Currently, large-aperture Fizeau-type interferometers and associated phase-shifting techniques have been widely studied for precision wavefront testing of flats and spheres [6,7,8], mainly used for applications such as interferometric wavefront testing, near-infrared intermediate frequency transmission capability, and system polarization aberration research [9,10].

However, high-precision testing of ultra-large-aperture flats faces two interrelated challenges. First, as the aperture increases beyond Φ600 mm, the absolute surface figure must be separated from the systematic errors of the reference optics, which requires dedicated absolute testing methods such as rotation–translation algorithms. Second, and more critically for large apertures, the optical element undergoes gravitational deformation that introduces stress-induced surface errors on the order of $10^{-2}\lambda$ or higher [11]. This deformation is pose-dependent: any rotation or translation of the test flat will alter the stress distribution and change the measured surface figure. Therefore, achieving absolute testing for ultra-large apertures requires not only a suitable algorithm but also a support structure that maintains negligible and repeatable stress during multi-pose measurements. The lack of such an integrated solution has become a major bottleneck in the production and testing of high-precision large-aperture optical elements [12,13].

Several methods have been proposed for large-aperture interferometric testing. Miao et al. [14] and Zhu et al. [15] developed Φ600 mm Fizeau interferometers with advanced phase-shifting techniques, but these systems do not address absolute testing or gravitational deformation. For the Φ800 mm scale, Ma et al. [16] demonstrated a three-flat absolute testing method; however, the stress-induced astigmatism had to be removed from the final results post hoc, indicating that the support structure itself did not fully suppress gravitational deformation during multi-pose operations. A method that integrates absolute testing capability with in situ stress compensation remains lacking.

To address these challenges, an ultra-large-aperture interferometric testing technique is proposed that combines the two-plate shearing absolute mutual testing method with a novel micro-stress support technology. The key innovation lies in the micro-stress support structure, which reduces pose-dependent surface deformation by three orders of magnitude compared to conventional sling suspension, ensuring that the gravitational deformation remains below λ/150 and is repeatable across different poses. This enables the rotation–translation absolute testing algorithm to be reliably applied at the Φ800 mm scale, where conventional support methods would introduce unacceptable stress variations during multi-pose measurements. The proposed technique is experimentally validated on a Φ800 mm flat, achieving PV = 0.0923λ and RMS = 0.0114λ with high repeatability. Compared to previous absolute test technologies [17,18,19], this technique offers higher precision and is broadly applicable for real-time testing of ultra-large-aperture planes. The technique is also user-friendly and significantly enhances testing efficiency.

## 2. Principle

### 2.1. Optical Design

**Figure 1:**
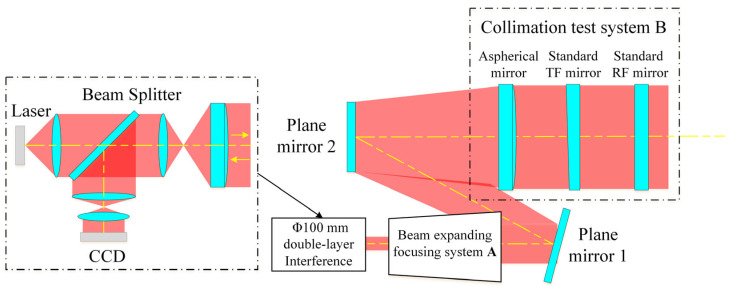
Optical path design of the Φ800 mm ultra-large-aperture digital laser standard plane interferometric testing device.

The Φ800 mm ultra-large-aperture digital laser standard plane interferometric testing device primarily consists of three parts: a horizontal Φ100 mm double-layer interferometric testing unit, an expansion and focusing system A, and a collimation adjustment system B. The optical path design is shown in Figure 1. This device uses a semiconductor laser source for illumination, with an average output wavelength stabilized at 632.8 nm and an output power of 5.4 mW. The beam emitted from the laser source first passes through the expansion and focusing system A, where it is converted into a converging beam, and then enters the collimation adjustment system B. This system precisely adjusts and aligns the optical path to achieve high-precision testing of large-aperture optical elements.

To ensure the testing accuracy of this device, the key lies in the selection of materials, processing technology, and testing methods for the standard TF and the standard RF. The interferometric testing unit utilizes a CCD with 2304 pixels × 2304 pixels, providing high-resolution detection capabilities. These design choices collectively ensure the overall performance and testing accuracy of the system. To retrieve the phase information from the interferograms with high accuracy, the standard Hariharan five steps phase-shifting algorithm [20] is employed. This algorithm is selected for its robustness against phase-shift calibration errors. Subsequently, the wrapped phase map is resolved into a continuous wavefront using a quality-guided phase unwrapping algorithm, ensuring reliable surface shape reconstruction.

### 2.2. Standard Flat Accuracy Guarantee

**Figure 2:**
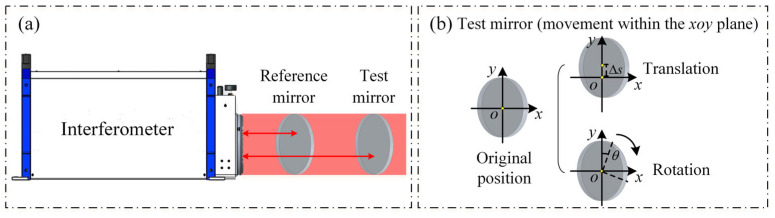
Diagram of the two-plate shearing absolute mutual testing method. (**a**) Specific structure; (**b**) test flat adjustment method.

To ensure the surface precision of the standard TF and the standard RF, this device employs a two-plate shearing absolute mutual testing method. This method uses two standard flats, including a reference flat and a test flat. By keeping the reference flat fixed while moving and rotating the test flat, the absolute surface shape distribution of both the reference and test mirrors can be achieved, as illustrated in Figure 2.

The method combines rotation and translation to decouple the reference flat error $W_{ref}\left(x,y\right)$ and the test flat error $W_{test}\left(x,y\right)$ from the measured wavefront $T\left(x,y\right)$. In practice, two standard flats are involved: one serves as the reference flat and remains fixed, while the other serves as the test flat and is sequentially rotated and laterally translated. By processing the resulting measurement set, the absolute surface figure distributions of both the reference and test flats can be retrieved, as illustrated in Figure 2. Let $T\left(x,y\right)$ denote the detected wavefront, which is the superposition of the two surfaces:

(1) $$T(x,y)=W_{ref}(x,y)+W_{test}(x,y)$$

Based on the spatial frequency characteristics, the test flat error $W_{test}\left(x,y\right)$ can be mathematically decomposed into a rotationally symmetric component $W^{s}\left(x,y\right)$ and a rotationally asymmetric component $W^{as}\left(x,y\right)$:

(2) $$W_{test}(x,y)=W^{s}(x,y)+W^{as}(x,y)$$

To isolate these components, the algorithm proceeds in two stages: rotation (to solve for $W^{as}$) and translation (to solve for $W^{s}$).

Step 1: We define a rotation sequence $Q_{i}\;(0\;\leq i\;\leq N-1$) representing the measurements taken when the test flat is rotated by N discrete angles with an increment of Δθ. The first three terms of this sequence are as follows:

(3) $$\begin{array}{c} Q_{0}=W_{ref}(x,y)+{\left[W_{test}(x,y)\right]}_{\theta }^{0}\\ Q_{1}=W_{ref}(x,y)+{\left[W_{test}(x,y)\right]}_{\theta }^{\Delta \theta }\\ Q_{2}=W_{ref}(x,y)+{\left[W_{test}(x,y)\right]}_{\theta }^{2\Delta \theta } \end{array}$$

where the operator ${\left[\cdot \right]}_{\theta }^{k\Delta \theta }$ denotes the coordinate rotation. Since $W_{\mathrm{ref}}$ is stationary, it remains invariant under the rotation operation ${\left[\cdot \right]}_{\theta }^{k\Delta \theta }$. The average rotation sequence $\left\{Q_{i}\right\}$ is defined as

(4) $$\bar{Q}(x,y)=\frac{1}{N}\sum_{i=0}^{N-1}Q_{i}(x,y)$$

In the ideal case of continuous-angle averaging, the rotationally asymmetric components vanish due to the orthogonality of angular harmonics, leaving only the rotationally invariant terms. However, for a discrete set of N equally spaced rotations, harmonic components whose azimuthal orders satisfy m=kN (k=1,2,…) may not be fully suppressed by the averaging. We denote the remaining N-fold rotationally symmetric residual by $W^{\left(N\right)}(x,y)$. Therefore, the average measurement can be written as

(5) $$\bar{Q}(x,y)=W_{\mathrm{ref}}(x,y)+W^{s}(x,y)+W^{\left(N\right)}(x,y)$$

Subtracting $\bar{Q}$ from the initial measurement $Q_{0}$ cancels both $W_{\mathrm{ref}}$ and $W^{s}$, yielding

(6) $$Q_{0}(x,y)-\bar{Q}(x,y)=W^{as}(x,y)-W^{\left(N\right)}(x,y)$$

When $W^{\left(N\right)}(x,y)$ is sufficiently small, the asymmetric component can be approximated by

(7) $$W^{as}(x,y)\approx Q_{0}(x,y)-\bar{Q}(x,y)$$

In this case, $W^{\left(N\right)}$ mainly introduces a small leakage between the retrieved symmetric and asymmetric components, and its influence on the final reconstruction accuracy can be neglected.

Step 2: To solve the rotationally symmetric term $W^{s}$, which cannot be detected by rotation, a translation operation is performed. The test flat is shifted by a distance Δx. The measurement result $T_{shift}$ is as follows:

(8) $$T_{shift}(x,y)=W_{ref}(x,y)+W_{test}(x-\Delta x,y)$$

Subtracting the shifted measurement from the original measurement $Q_{0}$ cancels the reference error $W_{ref}$, resulting in the difference wavefront of the test flat:

(9) $$\Delta W_{test}=Q_{0}(x,y)-T_{shift}(x,y)=W_{test}(x,y)-W_{test}(x-\Delta x,y)$$

Substituting the decomposition from Equation (2) into Equation (9), the total difference can be separated into symmetric and asymmetric differences:

(10) $$\Delta W_{test}=[W^{s}(x,y)-W^{s}(x-\Delta x,y)]+[W^{as}(x,y)-W^{as}(x-\Delta x,y)]$$

Since $W^{as}$ has already been determined in Step 1, its difference term (the second bracket in Equation (8)) is known. We can thus isolate the difference of the symmetric component $\Delta W^{s}$:

(11) $$\Delta W^{s}=\Delta W_{test}-[W^{as}(x,y)-W^{as}(x-\Delta x,y)]$$

Finally, the rotationally symmetric wavefront $W^{s}\left(x,y\right)$ is reconstructed from the finite difference data $\Delta W^{s}$ using the Zernike polynomial fitting method. The absolute surface shape of the test flat is then obtained as $W_{test}=W^{s}+W^{as}$, and the reference flat error is derived as $W_{ref}=\bar{Q}-W^{s}$.

### 2.3. Micro-Stress Support Technology

**Figure 3:**
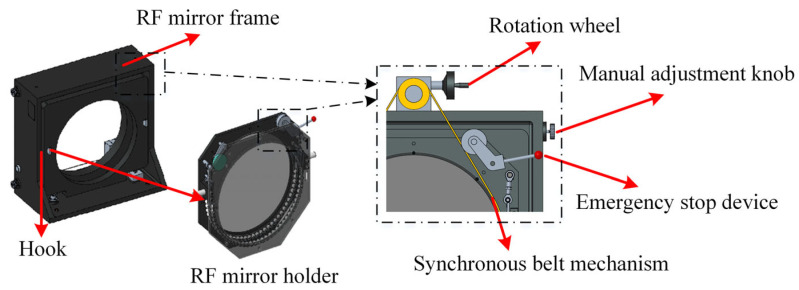
Detailed structure of the micro-stress support technology.

In the principle described in Section 2.2, it is necessary to precisely rotate the standard RF while ensuring that the element remains stable in all directions during rotation and minimizing stress birefringence effects caused by its own weight. To achieve this, micro-stress support technology is proposed. This technology employs a bi-axial bearing system, an adjustable-length connection mechanism, and a synchronous belt tensioning assembly to provide stress compensation without envelope stress. The structure is shown in Figure 3. The standard flat is mounted by hanging it on hooks attached to the flat frame. The flat frame is hung on the flat holder, and the tensioning wheel adjustment lever is pushed to bring the synchronous belt into contact with the flat. This setup is used to adjust the flat rotation, observe the value changes on the display, and achieve precise rotation.

To ensure precision during rotation, a rotary encoder with 262,144 lines and an accuracy of less than 0.01° is selected. The encoder’s wheel has an outer diameter of Φ100 mm, and the flat’s outer diameter is Φ830 mm. The rotation precision of the flat is 0.0012°, which is less than 0.1°. The encoder’s wheel is in direct contact with the flat, ensuring accurate measurement of the flat’s rotation angle and avoiding transmission errors that could arise from mounting on the drive mechanism.

**Figure 4:**
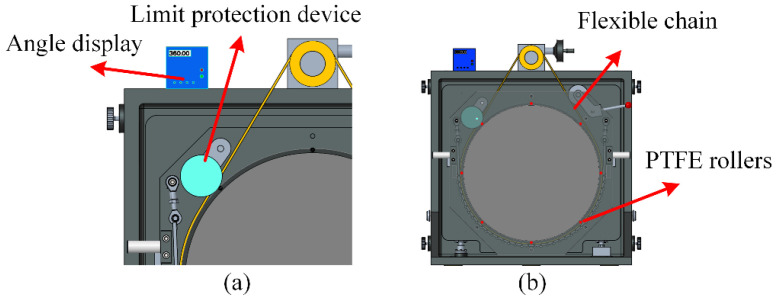
(**a**) Rotation precision control structure. (**b**) Normal direction stability control structure.

The synchronous belt pulley has a diameter of Φ100 mm, and the flat’s outer diameter is Φ830 mm, resulting in a transmission ratio of i = 8.3. With a gearbox reduction ratio of i = 60, calculations show that when the handwheel rotates one full turn, the flat rotates by 0.73°, ensuring the high-precision adjustment required for the flat. Additionally, a flexible chain and PTFE rollers ensure that the flat remains in the same position under its own weight. PTFE screws at the end faces restrict the flat’s displacement, ensuring that the flat does not deviate in the normal direction. The structure is shown in Figure 4. This setup maintains the stability of the standard flat’s center and the optical surface’s normal direction during rotation.

## 3. Results

### 3.1. Alignment and Initial Null Adjustment

**Figure 5:**
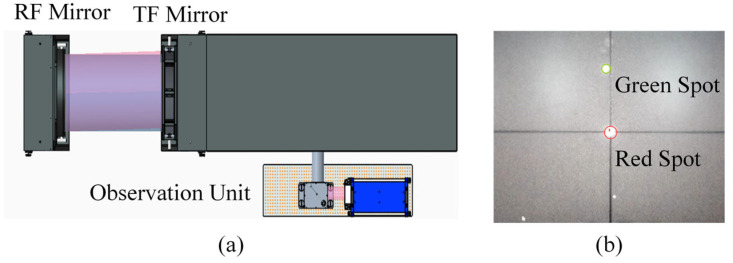
(**a**) Schematic layout of the alignment configuration. (**b**) Example image of the observation plane showing the green spot (RF) and the red spot (TF).

Before the absolute mutual testing, the system is aligned to an initial null condition. As shown in Figure 5a, the RF mirror is kept fixed, while the TF mirror is mounted on the motion stage for subsequent rotation and translation. An observation unit is used to monitor the autocollimation spots from the two mirrors. As illustrated in Figure 5b, the RF and TF spots are indicated by green and red markers, respectively. The relative tip/tilt between the two mirrors is iteratively adjusted until the two spots overlap on the observation plane, which defines the null state by minimizing the residual angular misalignment. After the overlap is achieved, the TF mirror is returned to the nominal position, and the null is rechecked to ensure repeatability. The subsequent rotation–translation measurements are then performed with this null configuration as the reference.

### 3.2. Standard Mirror Fabrication

**Figure 6:**
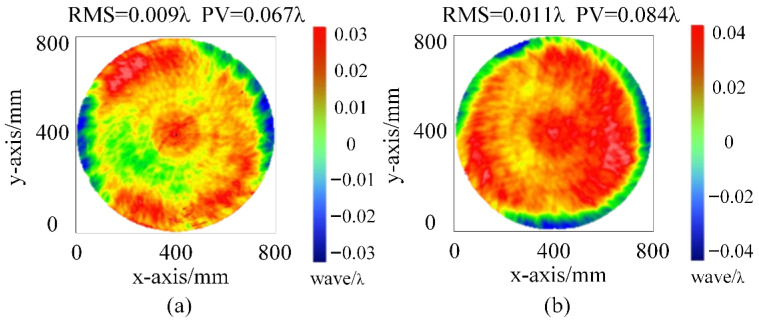
Measurement results of the standard TF and standard RF using the ZYGO interferometer: (**a**) measurement results of the standard TF; (**b**) measurement results of the standard RF.

In this experiment, we first fabricate the standard TF and the standard RF. The standard TF is made of fused silica and processed through ring polishing, large tool, and small tool magnetorheological finishing. After processing, the surface shape of the standard TF is measured using a 32″ ZYGO interferometer, yielding Peak-to-Valley (PV) and Root Mean Square (RMS) values of 0.067λ and 0.009λ, respectively, as shown in Figure 6. The standard RF, made of microcrystalline material, undergoes the same processing steps. The surface shape of the standard RF is also measured with a 32″ ZYGO interferometer, resulting in PV and RMS values of 0.084λ and 0.011λ, respectively, as shown in Figure 6.

**Figure 7:**
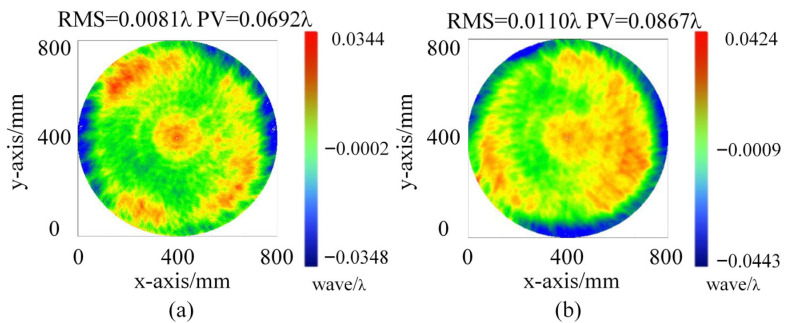
Measurement results of the standard TF and standard RF using the two-plate shearing absolute mutual testing method: (**a**) measurement results of the standard TF; (**b**) measurement results of the standard RF.

Next, the fabricated standard is installed in the device and tested using the two-plate shearing absolute mutual testing method described in Section 2.2. The results, shown in Figure 7, indicate that the surface shape PV and RMS values for the standard TF are 0.0692λ and 0.0081λ, respectively, while for the standard RF, the PV and RMS values are 0.0867λ and 0.0110λ, respectively. Comparison with the 32″ ZYGO interferometer results show a high degree of consistency, with minimal error.

### 3.3. Verification of Micro-Stress Support Technology

**Figure 8:**
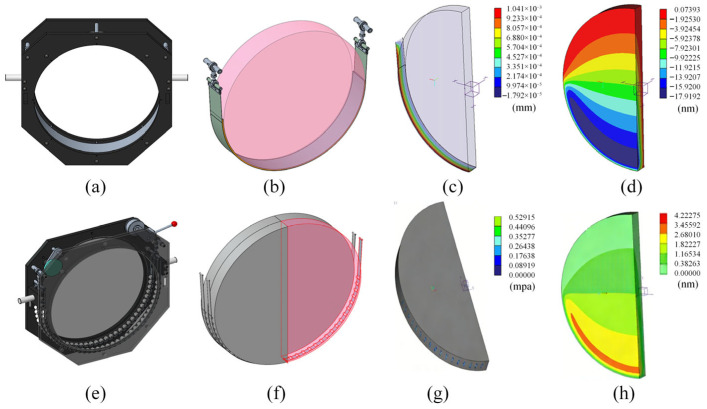
(**a**) CAD model of the sling-support frame. (**b**) Sling-based suspension configuration of the flat. (**c**) Displacement distribution under gravity for the sling support. (**d**) Resulting surface shape change of the flat under the sling support. (**e**) CAD model of the proposed micro-stress support structure. (**f**) Planar layout of the micro-stress support configuration. (**g**) Stress distribution in the support/contact region. (**h**) Resulting surface shape change of the flat under gravity with the proposed micro-stress support.

In practical applications, true free-boundary conditions do not exist; therefore, a flexible micro-stress support structure is required to approximate a free boundary for large-aperture optical elements during absolute testing involving rotation and translation. To highlight the necessity of the proposed support and quantify its benefit, we first compare it with a conventional sling-based suspension. Figure 8a–d show the sling-support configuration and its gravity response: the maximum displacement reaches approximately $1.0\times 10^{-3}$ mm as shown in Figure 8c. In contrast, the proposed rotation-point micro-stress support shown in Figure 8e–h reduces the maximum displacement to about $4.0\times 10^{-6}$ mm. Correspondingly, the gravity-induced surface shape change satisfies the λ/150 criterion.

**Figure 9:**
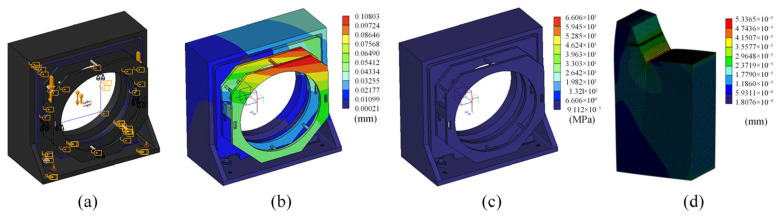
(**a**) Simplified CAD model and boundary-condition setup of the overall frame in Simulate. (**b**) Displacement distribution of the overall frame under gravity loading. (**c**) Von Mises stress distribution of the overall frame under gravity loading. (**d**) Displacement distribution of the hook component under the equivalent load.

To further address concerns regarding residual clamping force and the mechanical robustness of the supporting hardware, additional FEM analyses were conducted for the overall frame and the hook assembly, as summarized in Figure 9. The displacement distribution of the frame under gravity loading is shown in Figure 9b, where the maximum displacement is approximately 0.108 mm. The corresponding von Mises stress distribution is presented in Figure 9c, with a maximum stress of about 66 MPa. The peak deformation occurs at the upper corner due to the L-shaped load path and is not concentrated at the mirror–support interface. For the hook component, the maximum displacement is only $5.34\times 10^{-5}\;mm$, indicating negligible compliance in the constraint/contact region and supporting the “micro-stress” claim from a structural perspective. Together with the measured motion performance of the calibration mechanism, these results confirm that the support hardware provides sufficient stiffness and repeatability for the rotation–translation absolute mutual testing procedure.

### 3.4. Planar Surface Shape Testing

**Figure 10:**
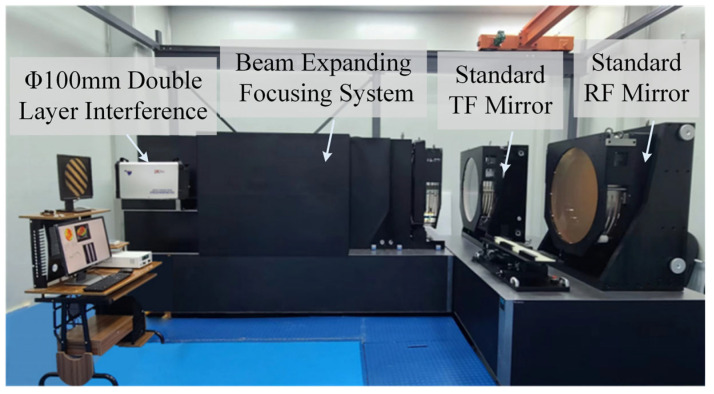
Structure of the Φ800 mm ultra-large-aperture digital laser standard plane interferometric testing device.

By combining the methods described earlier, the Φ800 mm ultra-large-aperture digital laser standard plane interferometric testing device is successfully constructed. This device is independently developed by Chengdu Tyggo Photoelectric Technology Co., Ltd., featuring an effective aperture of Φ800 mm for testing output. To guarantee high-precision measurement stability for such a large aperture, the testing system is in a Class 1000 cleanroom with controlled temperature (20 ± 0.5 °C) and humidity. To suppress environmental vibrations, the device is supported by a pneumatic vibration isolation platform with a supply pressure of 0.6 MPa. Additionally, the random noise caused by air turbulence is effectively minimized through the averaging process of multiple measurements during the rotation and translation steps. The structure of the device is shown in Figure 10.

**Figure 11:**
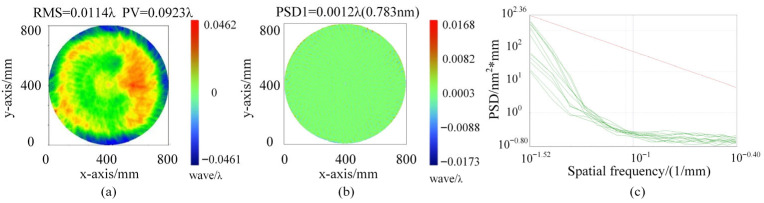
Measurement results of the Φ800 mm planar element: (**a**) Surface shape precision; (**b**) PSD1 value for the mid-frequency band; (**c**) mid-frequency PSD1 evaluation curve.

Using this device, the Φ800 mm planar element under examination is tested. During the absolute mutual testing, the test flat is laterally translated by ±60 mm in both the x- and y-directions, achieving a positioning accuracy of approximately 0.1 mm. Subsequently, the test flat is rotated in four discrete steps with an angular increment of $10^{\circ }$, maintaining a rotation accuracy of approximately 6 arcmin. The measured surface shape precision is PV = 0.0923@632.8 nm and RMS = 0.0114@632.8 nm, as shown in Figure 11a. To quantitatively evaluate the surface shape error in the spatial frequency domain, the Power Spectral Density (PSD) of the distorted wavefront was first calculated. Following the protocols of the National Ignition Facility (NIF), the spectral data is categorized into low-spatial-, mid-spatial-, and high-spatial-frequency bands. In this study, we specifically focused on the mid-spatial-frequency (MSF) band, defined as the range from $0.03\;{mm}^{-1}$ to $0.4\;{mm}^{-1}$. Figure 11b illustrates the isolated surface error map within this MSF band, which corresponds to an RMS value of 0.777 nm. As shown in Figure 11c, the evaluated one-dimensional PSD (PSD1) curve lies significantly below the control line (the maximum allowable acceptance threshold), indicating that the surface shape error in the critical mid-frequency band is minimal.

To further validate the stability and reliability of the testing system, a repeatability test was conducted on the Φ800 mm planar element. Ten consecutive measurements were performed under the same environmental conditions. The variations in surface shape parameters are detailed in Table 1. The results show that the mean PV value is 0.0923λ with a standard deviation (STD) of 0.0012λ, and the mean RMS value is 0.0114λ with a standard deviation of 0.0001λ. These low standard deviation values confirm that the proposed method possesses high measurement repeatability and robustness against environmental noise.

## 4. Discussion

**Figure 12:**
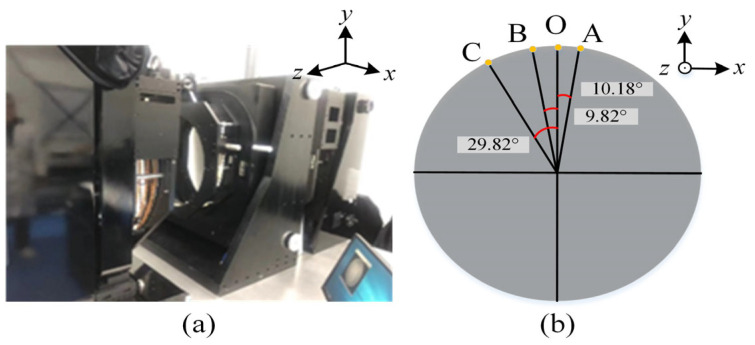
(**a**) Rotational fixture assembly; (**b**) adjustment path for standard RF.

To evaluate the mechanical stability of the proposed micro-stress support structure during practical operation, both rotation and translation of the test flat are analyzed experimentally and numerically. As described in Section 2.3, it is crucial to ensure that the standard RF does not undergo significant surface shape changes during rotation, which could affect the experimental results. Rotation experiment is first conducted using a 610 mm flat installed on the micro-stress support assembly. The rotational fixture assembly, shown in Figure 12a, is manufactured to test the standard RF. The adjustment path involves rotating from point O to B by 9.82 degrees, then back from B to O by 9.82 degrees, from O to A by 10.18 degrees, and finally from A to C by 40 degrees, as illustrated in Figure 12b.

**Figure 13:**
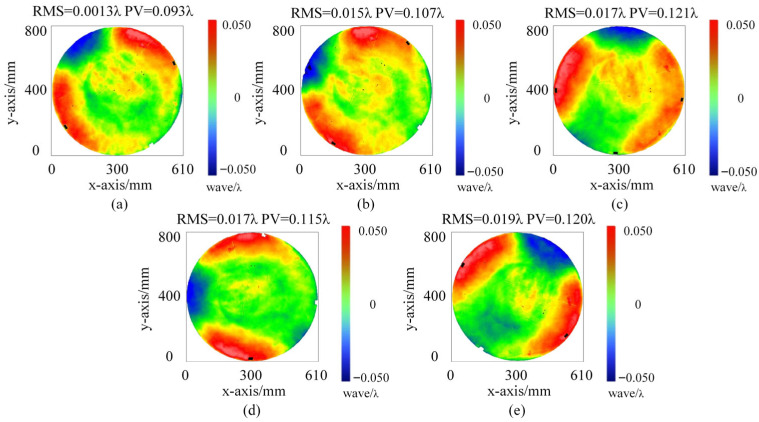
Surface shape measurement results in different rotation positions. (**a**) Surface figure measurement at the original position O before rotation. (**b**–**e**) after four sequential ro-tations.

After each rotation, the surface shape of the standard flat is measured using a ZYGO interferometer. The results are presented in Figure 13a–e. The RMS values of the measurements are compiled in Table 2, and the absolute errors after each rotation are calculated. Observations show that the maximum RMS absolute error is 3.796 nm, which meets the testing criteria. These results confirm the effectiveness of the micro-stress support compensation structure.

In addition to rotation, the translation of the test flat may also introduce surface figure variations. In this work, the translation is achieved by repositioning the mirror frame onto different pre-machined hook locations on the support bracket, rather than using a continuous guideway. As described in Section 2.3, the hook positioning holes are precision-machined with pin-locating features. Five sets of hook positions are available on the bracket, enabling lateral translations of ±60 mm in both horizontal and vertical directions.

**Figure 14:**
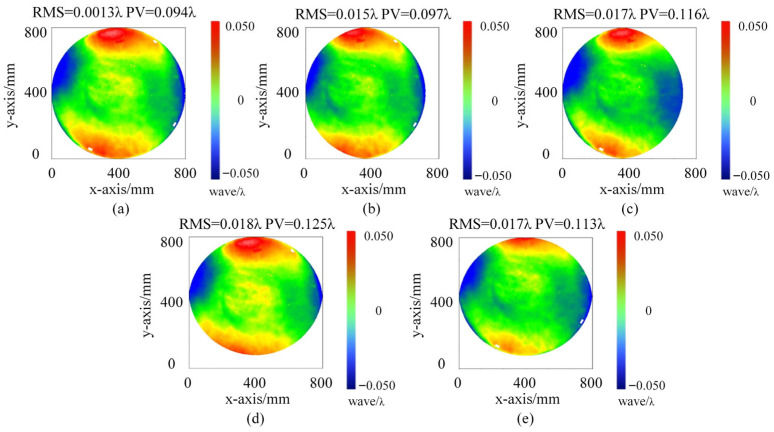
Surface figure measurement results at different translation positions: (**a**) original position O; (**b**–**e**) after four sequential translations.

To evaluate the surface figure stability during translation, the standard flat was measured at the original position and after four sequential translations. The measurement results are presented in Figure 14. At the original position, the surface figure shows RMS = 0.013λ and PV = 0.094λ. After the four translations, the RMS values range from 0.015λ to 0.018λ, and the PV values range from 0.097λ to 0.125λ. The maximum RMS deviation from the original position is approximately 0.005λ, which remains well within the λ/150 tolerance. The small deviations are attributed to slight differences in the contact conditions between the hooks and the mirror frame at different positions.

Nevertheless, a perfectly ideal translation is not always guaranteed, and a small parasitic tip/tilt may still arise from hook seating variations or minor angular play during repositioning. Such minute angular deviations tend to couple into the interferometric wavefront primarily as low-order components, most notably affecting the Zernike fourth term (defocus/power), and may bias the absolute reconstruction if left unobserved. In this work, the observation unit used for initial null adjustment in Section 3.1 provides an autocollimation-based angular reference and can be directly leveraged as an in situ angle-monitoring channel during translation, a strategy similar to the relative tilt measurement concept employed by Zhai et al. [21] for absolute flat testing. By tracking the relative spot motion on the observation plane, the instantaneous tilt can be monitored and used for quality control by rejecting non-ideal translation states, thereby mitigating power-related errors. The translation measurement results in Figure 14 show an RMS stability of 0.005λ across multiple positions. This high stability explicitly confirms that the translation induced parasitic tilt has been effectively suppressed by the monitoring technique, demonstrating the excellent performance of the actual defocus correction.

Taken together, both the rotation experiment and the translation measurement confirm that the mechanical influences introduced during the adjustment process, whether from rotation or translation, do not accumulate to a level that affects the accuracy of the absolute measurement. The proposed micro-stress support structure therefore provides sufficient mechanical stability to ensure reliable high-precision testing of ultra-large-aperture planar elements.

## 5. Conclusions

In conclusion, a micro-stress support technology has been designed and validated in this paper to address the critical challenge of gravitational deformation in large-aperture metrology. This support structure constitutes the primary contribution of the work, enabling repeatable multi-pose measurements that allow the rotation–translation absolute testing algorithm to be reliably applied at the Φ800 mm scale. Experimental results for an 800 mm planar element yielded a PV value of 0.0923λ and an RMS value of 0.0114λ at a wavelength of 632.8 nm. The high consistency with 32″ ZYGO interferometer measurements confirms the precision and stability of the proposed support technology. Furthermore, this work demonstrates a viable solution for the high-precision detection of large-aperture planar elements, proving its broad applicability. Future research will focus on further optimizing the technique to accommodate larger sizes and more complex shapes of optical elements.

## Figures and Tables

**Table 1:** Repeatability measurement results of the Φ800 mm planar element.

Measurement No.	PV@632.8 nm	RMS@632.8 nm
1	0.092	0.013
2	0.093	0.013
3	0.093	0.012
4	0.095	0.012
5	0.092	0.013
6	0.093	0.013
7	0.095	0.014
8	0.092	0.012
9	0.092	0.013
10	0.092	0.014
Mean	0.093	0.013
STD	0.001	0.0007

**Table 2:** The measurement results at different rotation positions.

Position	O	O→B	B→O	O→A	A→C
RMS@632.8 nm	0.013	0.015	0.017	0.017	0.019
Absolute Error/nm	/	1.265	2.531	2.531	3.796

## Data Availability

The original contributions presented in this study are included in the article. Further inquiries can be directed to the corresponding authors.

## References

[1] Zhang Y., Wu Y., Fan B. (2013). Theoretical and experimental study of a catadioptric compensator for an aspheric surface. Appl. Opt..

[2] Lightsey P.A., Atkinson C., Clampin M., Feinberg L.D. (2012). James Webb Space Telescope: Large deployable cryogenic telescope in space. Opt. Eng..

[3] Gensemer S., Gross M. (2015). Figuring large optics at the sub-nanometer level: Compensation for coating and gravity distortions. Opt. Express.

[4] Burge J.H., Kot L.B., Martin H.M., Zhao C., Zobrist T. (2006). Optical metrology for the 8.4 m diameter mirror segments for the 25m Giant Magellan Telescope. Proceedings of the Optical Fabrication and Testing.

[5] Bray M., Roussel A. (1996). Using first principles in the specifying of optics for large high-power lasers (I): Application to the Megajoule laser (LMJ). Proceedings of the Specification, Production, and Testing of Optical Components and Systems.

[6] Wang Y.T., Chen L., Chen J., Zhang Z.Y., Lu K. (2020). Distortion correction research by gravity for 300-mm-aperture flat based on multi-point adjustable support. J. Appl. Opt..

[7] Zhou Y., Liu S., Lu Q., Zhu R., Vdovine G. (2020). In situ absolute surface metrology for a 600 mm aperture interferometer. Opt. Lasers Eng..

[8] Zhao Z.L., Liu M., Chen L.H., Fu Y.G. (2019). Φ200 mm long focal length spherical interference test and equipment. Chin. Opt..

[9] Guo R.H. (2013). Research on Key Techniques and Applications of the Near-Infrared Large Aperture Wavelength-Tuning Interferometer. Ph.D. Thesis.

[10] Yue Y. (2021). A Method of Collimating Wavefront Testing by Corner-cube-reflector. Master’s Thesis.

[11] Yang L.J., Xing T.W., Fen J. (2013). A compensation method of large aperture lens for gravity deformation. Proceedings of the 7th International Symposium on Advanced Optical Manufacturing and Testing Technologies: Large Mirrors and Telescopes.

[12] Wang Y., Chen L., Hu C., Peng X., Zhang Y. (2021). Iterative deformation calibration of a transmission flat via the ring point support on a 300-mm-aperture vertical Fizeau interferometer. Opt. Express.

[13] Evans C.J., Davies A.D. (2013). Certification, self-calibration and uncertainty in optical surface testing. Int. J. Precis. Technol..

[14] Miao X., Yu Y., Li A., Farrell C.T., Zou X., Lehan J.P., Kuchel M. (2021). Optical phase-shifting methods based on low coherence laser for large aperture Fizeau interferometer. Opt. Lasers Eng..

[15] Zhu W., Chen L., Yang Y., Shi T., Peng X., Bai J. (2018). 600-mm aperture simultaneous phase-shifting Fizeau interferometer. Opt. Laser Technol..

[16] Ma Z., Chen L., Ma J., Peng X., Sun Z. (2024). Absolute tests of three flats for interferometer with 800 mm aperture. Opt. Express.

[17] Zhang Y.W., Su D.Q., Sui Y.X., Xu J.Y., Cui Y.F. (2014). Absolute Testing of Rotationally Asymmetric Surface Deviation with the Method of Rotation-Averaging and Compensation. Chin. J. Lasers.

[18] Sun W.Q., Chen L., Xu C. (2010). Absolute measurement of planarity with two-flat test. J. Southeast Univ..

[19] Song W.H., Wu F., Hou X., Xing T. (2013). Absolute measurements of flats with the method of shift rotation. Opt. Rev..

[20] Hariharan P., Oreb B.F., Eiju T. (1987). Digital phase-shifting interferometry: A simple error-compensating phase calculation algorithm. Appl. Opt..

[21] Zhai D., Chen S., Peng X. (2019). Absolute flat test using rotated and multi-shifted maps with relative tilt measurement. Opt. Lasers Eng..

